# Antireflux mucoplasty with the reopenable clip-over-the-line method for refractory gastroesophageal reflux disease

**DOI:** 10.1055/a-2587-8838

**Published:** 2025-05-26

**Authors:** Jiawei Lin, Li Fan, Shuangshuang Lu, Min Lin

**Affiliations:** 1 599923 Department of Gastroenterology, Changzhou Second Peopleʼs Hospital of Nanjing Medical University, Changzhou, China

We report here the case of a 57-year-old woman who was referred to our hospital with suspected reflux esophagitis following epigastric and retrosternal discomfort for 1 year. She underwent barium fluoroscopy of the upper gastrointestinal tract, which revealed lower esophageal wall irregularity and gastroenteritis. High-resolution esophageal manometry showed a resting lower esophageal sphincter pressure of 9.2 mmHg, and upper endoscopy showed erosive esophagitis with a hiatal hernia of Hill’s flap grade III ( Fig. 1 ). Reflux Disease Questionnaire results suggested a total score of 20. All of these results met the criteria for antireflux mucoplasty 1 .

**Fig. 1:**
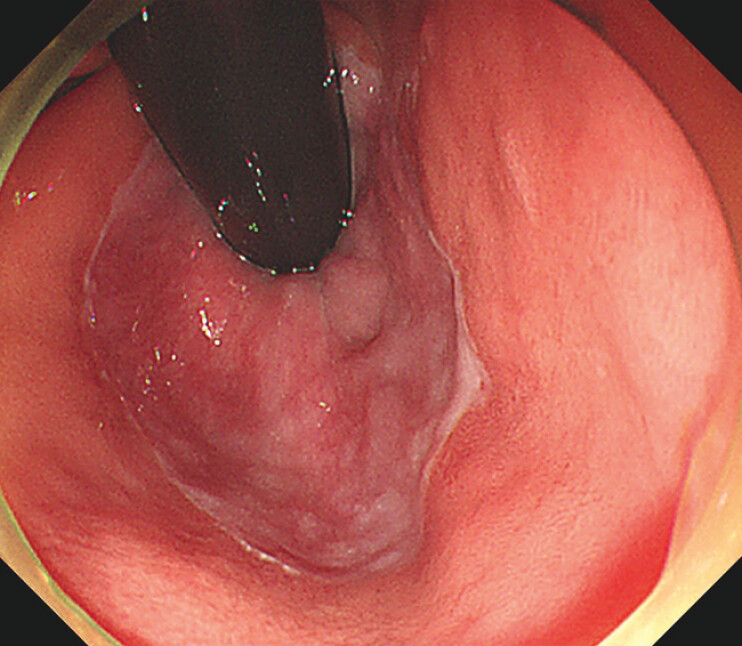
The Hill’s flap grade was classified as III before antireflux mucoplasty.

The procedure involved injecting saline mixed with indigo carmine dye and epinephrine into the submucosa along the lateral aspect of the marked points until the mucosa was sufficiently elevated. Cap-assisted endoscopic mucosal resection with submucosal injections was repeated three times to remove the mucosa from half of the circumference of the cardia, preserving the mucosa on the other half on the greater curvature side ( Fig. 2 ). Hemostasis was achieved with thermal coagulation.

**Fig. 2:**
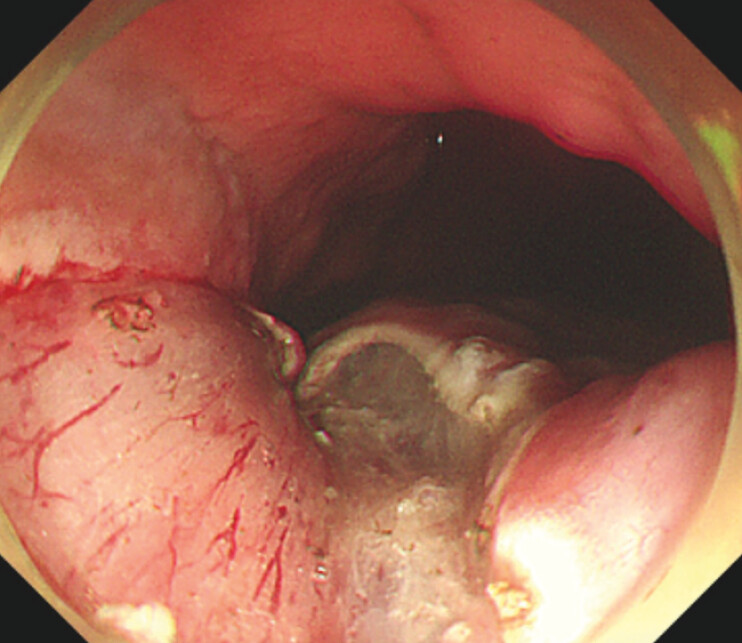
The mucosal defect after mucosectomy.

Closure of the mucosal defects was performed using a novel method, the reopenable clip-over-the-line method (ROLM), which allows continuous traction with a line during closure with an endoscopic clip ( Fig. 3 , Video 1 ) 2 .

**Fig. 3:**
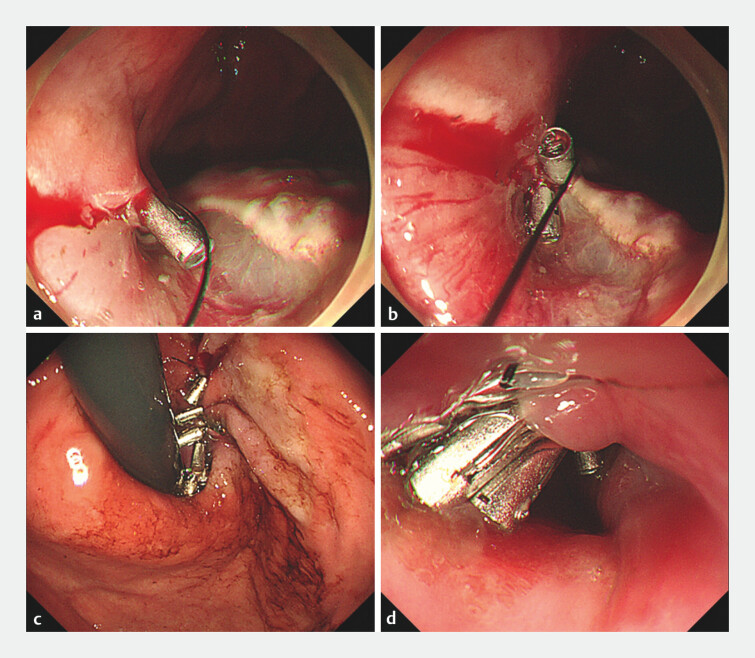
Defect closure using the reopenable clip-over-the-line method (ROLM). **a** The line was tied to the tooth of a reopenable clip, and the edge of the mucosal defect and muscle layer were simultaneously grasped using the reopenable clip. **b** The reopenable clip was used to grasp the mucosal defect edge and muscle on the opposite side of the first clip. **c, d** Complete closure of mucosal defects with repeated ROLMs.

Antireflux mucoplasty with the reopenable clip-over-the-line method for refractory gastroesophageal reflux disease.Video 1

A follow-up endoscopy 5 months post-procedure revealed a tightened hernia with an improvement in Hill’s flap grade from III to II ( Fig. 4 ). High-resolution esophageal manometry showed a lower esophageal sphincter pressure of 16.3 mmHg, which was within the normal range. The patient’s postoperative pain was mild and recovery was rapid. No adverse events were reported.

**Fig. 4:**
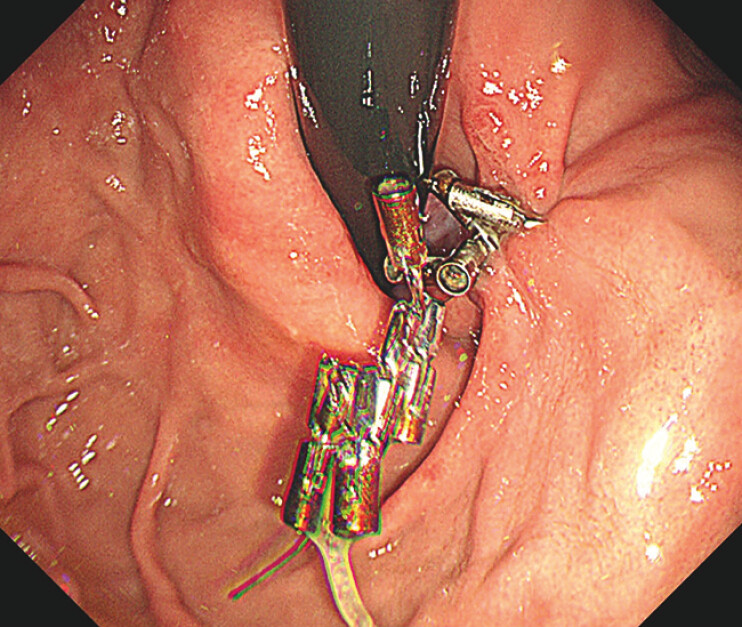
At the 5-month follow-up, the Hill’s flap grade had improved to grade II.

Antireflux mucoplasty assisted by ROLM can address some challenges of antireflux mucosal resection and antireflux mucosal ablation, including the slow onset of therapeutic effect and the risk of delayed bleeding in patients taking antithrombotic medications 3 . In addition, it facilitates continuous closure without the formation of submucosal dead space.

Endoscopy_UCTN_Code_TTT_1AO_2AJ

## Notes

Endoscopy E-Videos https://eref.thieme.de/e-videos
*E-Videos* is an open access online section of the journal *Endoscopy* , reporting on interesting cases and new techniques in gastroenterological endoscopy. All papers include a high-quality video and are published with a Creative Commons CC-BY license. Endoscopy E-Videos qualify for HINARI discounts and waivers and eligibility is automatically checked during the submission process. We grant 100% waivers to articles whose corresponding authors are based in Group A countries and 50% waivers to those who are based in Group B countries as classified by Research4Life (see: https://www.research4life.org/access/eligibility/ ). This section has its own submission website at https://mc.manuscriptcentral.com/e-videos .
